# Spin-Orbit Coupling Effects in Au 4f Core-Level Electronic Structures in Supported Low-Dimensional Gold Nanoparticles

**DOI:** 10.3390/nano11020554

**Published:** 2021-02-23

**Authors:** Smruti R. Sahoo, Shyue-Chu Ke

**Affiliations:** Department of Physics, National Dong Hwa University, Hualien 974301, Taiwan; smruti@gms.ndhu.edu.tw

**Keywords:** supported nanoparticles, gold nanoparticle-based catalysts, core-level electronic structure, X-ray photoelectron spectroscopy, Au 4f_7/2_-to-Au 4f_5/2_ peak intensity and linewidth ratios

## Abstract

Despite their many advantages, issues remain unresolved over the variability in catalytic activities in supported gold nanoparticle (AuNP)-based catalysts, which requires precise characterization to unravel the presence of any fine features. Herein, upon analyzing the Au 4f core-level spin-orbit components in many as-synthesized AuNP-based catalysts, we observed that like deviations in the Au 4f_7/2_ binding energy positions, both the Au 4f_7/2_-to-Au 4f_5/2_ peak intensity and linewidth ratios varied largely from the standard statistical bulk reference values. These deviations were observed in all the as-synthesized supported AuNPs irrespective of different synthesis conditions, variations in size, shape or morphology of the gold nanoparticles, and different support materials. On the other hand, the spin-orbit-splitting values remained almost unchanged and did not show any appreciable deviations from the atomic or bulk standard gold values. These deviations could originate due to alterations in the electronic band structures in the supported AuNPs and might be present in other NP-based catalyst systems as well, which could be the subject of future research interest.

## 1. Introduction

Progress in synthesis methods and advances in sophisticated characterization techniques have made it possible to engineer the surface morphology of various types of low-dimensional nanostructures. In turn, this has helped in understanding their novel physicochemical properties in a more detailed manner [1,2]. Recent interest has focused more on the application, postprocessing, and anchoring of nanoparticles (NPs) with different morphologies on different dielectric matrices in order to exploit the synergistic effects from both the nanoparticle surface and the underlying substrate materials [3,4]. These modifications ultimately result in novel structural, optical, electronic, and catalytic properties [5,6]. For instance, unlike chemically inert bulk gold, gold nanoparticles (AuNPs), when supported on various substrates, become catalytically active toward various organic reaction transformations and fuel-cell conversion-related applications [7,8,9]. In such cases, apart from the agglomeration minimization and stabilization of gold nanoparticles, it has been observed that the selection of suitable support materials often plays a dominant role in dictating the rate of CO oxidation, water–gas shift reactions [8,10,11], and selective oxidation or hydrogenation reactions [12]. The role of the support material is thus very crucial for specific catalytic reactions, thereby demanding the deliberate design of supports with specific functionalities [4]. Besides, metal-support interactions often play dominant roles in the wetting of the support material by the nanoparticle, greater adhesion of the nanoparticles with the support material, and charge transfer to/from metal nanoparticle and the support material, resulting in electron-rich metallic NPs/support materials, an interfacial bonding layer, and the enhancement or suppression of certain organic reactions [13]. Overall, catalytic activity and selectivity are highly influenced by an interplay from the nanoscale properties, such as particle shape, size, and morphology; strain from the substrate material, undercoordinated surface atoms at edges and vertices; characteristic defects; and the support material [14]. The selection of suitable support materials has surged with the emergence of graphene and many other two-dimensional materials [8,10,15,16]. Use of these wide variety of support materials is now being more intensively pursued over AuNPs alone, which promotes both the catalytically active and reactive nature of AuNPs to become more exciting areas of research [12]. The emergence of these two-dimensional materials also requires novel synthesis methods [17], different than conventional synthesis routes [18], to load gold nanoparticles in order to obtain the desired catalytic activities.

Despite all the advancements, the development of efficient catalysts poses several challenges, including the lack of robust design, problems like activity and selectivity, and variability in catalytic activities. Alterations in catalytic performance are often seen to be influenced by finetuning the redox and acid-base properties and the sizes of the active phases. Moreover, the nature of the support and size of the active phases controls the selectivity and activity of the reactant molecules [19]. To underpin the selected issues, the catalysts need to be thoroughly characterized to unravel any existing fine features present in the catalysts. Sensitivity at the atomic level is becoming an essential criteria to characterize NP-based catalyst systems, owing to the emergence of new and novel catalysts such as single-atom catalysts (SACs) and to the small amount of NP contents being used in the NP-based catalyst system [20,21]. In this regard, researchers have demonstrated the vast utility of X-ray Photoelectron Spectroscopy (XPS) in characterizing nanostructured materials, which can bring information from a depth similar to the sizes of the nanoparticles in a nondestructive manner. XPS has been extensively used to understand charge transfer to/from the nanoparticle/support [22], metal–support interaction [22], formation and measurement of Schottky barrier height [23], energy-band alignments [24], coordination number imperfection in the nanoparticle surface atoms, and catalytically active sites [25,26]. Besides, XPS has been used to investigate electronic, structural, and chemical changes and unravel active phases responsible for organic reaction transformations in many catalyst systems [27,28,29]. Although XPS provides a plethora of physicochemical information which can be both qualitative and quantitative, XPS has not yet been fully utilized in the characterization of complex nanoparticle surfaces [30].

One of such underutilized XPS feature was first discovered by Chenakin-Kruse on the deviation in the Au 4f_7/2_-to-Au 4f_5/2_ peak intensity and linewidth ratios from standard statistical values in TiO_2_ supported small gold nanoparticles [31]. Later, in an attempt to improve the XPS quantitation from nanostructured materials, we also observed similar deviations in Au 4f_7/2_-to-Au 4f_5/2_ peak intensity ratios from the standard statistical bulk values in TiO_2_-supported AuNPs [32]. With current interest being more inclined toward the use of various support materials, in this study, we selected a host of support materials, namely metal oxides, dichalcogenide, and nitrides for anchoring gold nanoparticles. These include TiO_2_, Al_2_O_3_, V_2_O_5_, NiO, Ti_2_O_3_, BN, and MoS_2_, respectively. Apart from gold nanoparticles, anisotropic gold nanorods were also anchored onto the selected host of support materials. This can further shed insights into whether particle anisotropy affects the Au 4f_7/2_-to-Au 4f_5/2_ peak intensity and linewidth ratios. Two different synthesis routes, namely the chemical impregnation and surface functionalization methods, were adopted to support nanoparticles and nanorods onto the selected supports. We made a careful analysis into the Au 4f core-level electronic structures in all these as-synthesized low-dimensional nanostructures under such different experimental conditions. The Au 4f_7/2_ binding energy position and Au 4f_7/2_-to-Au 4f_5/2_ peak intensity and linewidth ratios in all of the supported nanostructures were analyzed to evaluate their similarity or deviation from their bulk counterparts.

## 2. Materials and Methods

The syntheses of the various supported gold nanostructures used in this study are elaborated in the Electronic Supporting Information, pp S1–S3. In the chemical impregnation synthesis procedure, a suitable amount of HAuCl_4_ precursor and support material were mixed together in aqueous media and vigorously stirred in the dark at room temperature for 6 h. Second, ice-cold NaBH_4_ was used to reduce HAuCl_4_ to produce AuNPs, which were subsequently attached onto the support materials. The stirring was continued for another 4 h to consume all the sodium borohydride content present in the solution. The solution was then centrifuged and washed multiple times with deionized water in order to remove the chlorine residuals. Finally, the collected precipitates were oven-dried at 95 °C for 16 h. In the surface functionalization method, short anisotropic gold nanorods (AuNRs) were synthesized first following a 2-step seed-mediated approach. 2 two-step seed-mediated approach involves (1) the generation of small gold nanoparticle seeds, and (2) the preparation of a growth solution for the initiation of gold nanorod (AuNR) growth from the presynthesized seed particles. A typical synthesis procedure involves the mixture of an appropriate amount of CTAB (cetyltrimethylammonium bromide) and HAuCl_4_ in aqueous solution at room temperature, followed by the addition of ice-cold NaBH_4_ under rapid stirring condition. The stirring was continued further for 2 h to consume all of the sodium borohydride present in the solution. The growth solution was prepared by gently mixing an appropriate amount of CTAB and HAuCl_4_ in aqueous media, followed by the addition of ascorbic acid (AA). The use of ascorbic acid (AA) led to the observation of the yellow-colored solution turning transparent, indicating the reduction of Au^3+^ to Au^+^. For the formation of uniform anisotropic AuNRs with finetuned aspect ratios, a small number of Ag^+^ ions was added to the growth solution. Finally, a suitable amount of the presynthesized seeds particles was added to the growth solution to initiate the formation AuNRs with preferred aspect ratios.

The as-synthesized AuNRs were then purified by centrifugation to remove the excess amount of CTAB. For their support onto different substrates, both the AuNRs and the different support materials were functionalized with thioglycolic acid (TGA). After functionalization, excess amounts of TGA from the supported AuNRs were removed by further purification. The as-synthesized AuNR-based catalysts on different supports were then centrifuged and finally oven-dried at 95 °C for 16 h. The selected support materials used in both the chemical impregnation and surface functionalization synthesis routes were TiO_2_, Al_2_O_3_, V_2_O_5_, NiO, Ti_2_O_3_, BN, and MoS_2_, respectively.

The as-synthesized AuNRs/AuNPs supported on different substrates were characterized by Scanning Electron Microscopy (FESEM, Model: JSM 7000F, JEOL Ltd., Tokyo, Japan), Energy Dispersive X-ray Spectrometry (EDS, JEOL Ltd., Tokyo, Japan), and X-ray Photoelectron Spectroscopy (XPS, Thermo Scientific, Waltham, MA, USA), respectively. A Thermo-Scientific $K_{\alpha }$ X-ray Photoelectron Spectrometer employing monochromatic ${\mathrm{Al}\;K}_{\alpha }$ X-ray radiation at 1486.6 eV was used to acquire the high-resolution spectra from the as-synthesized catalyst samples. The spectra were acquiesced at a constant analyzer pass energy of 50 eV, with 0.025 eV step sizes. A low-energy electron flood gun was used to compensate the surface charging of the samples during operation. The spectrometer was calibrated using the inbuilt gold, silver, and copper standard samples. The uncertainty for binding energy measurements was checked by the position of a bulk Au 4f_7/2_ photoelectron line to 84.0 eV and a C1s photoelectron line at 284.8 eV, which was estimated to be ±0.02 eV.

Both the supported gold nanoparticle (AuNP)- and anisotropic gold nanorod (AuNR)-based catalyst materials were then subject to photoelectron spectroscopic analysis, particularly to reveal any changes in their respective core-level spin-orbit components. The Au 4f spin-orbit-splitting values (3.67 eV) were large enough to analyze each individual spin-orbit component separately in a typical high-resolution Au 4f XPS spectra. Furthermore, no plasmon or Auger peaks occurred in the Au 4f spectral region of interest, 92 (±1) − 79 (±1) eV, while employing the monochromated AlK_α_ radiation at 1486.6 eV to acquire the high-resolution spectra. The acquiesced Au 4f spectra from the catalysts were subject to a Shirley method of background subtraction procedure, with fixed binding energy endpoints. The individual spin-orbit components were first fitted with mixed Gaussian-Lorentzian (GL) types. The GL ratios were constrained initially to equal values. For a better fitting, the GL ratios were varied freely in a range from 20/80 to 1/99. All the parameters were finally optimized with slight asymmetry adjustments by a nonlinear least-square fitting procedure to yield a minimum χ^2^ value. It was observed that the final GL ratios remained around 15/90 in all spectra. The average values of binding energy peak position, peak intensity, and FWHM of the individual spin-orbit components were determined by fitting each spectrum 15 times. A standard t-test performed on the averaged values indicated a 95% confidence level, thereby implicating the significance of the fitting results.

## 3. Results and Discussions

**Figure 1:**
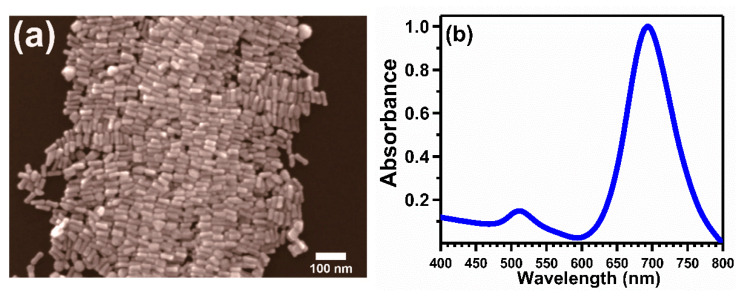
CTAB (Cetyl trimethylammonium Bromide)—assisted short anisotropic gold nanorods (AuNRs) synthesized by a two-step seed-mediated approach ((**a**), scale bar: 100 nm). The optical absorbance spectra from the as-synthesized short AuNRs display two plasmon bands corresponding to the transverse and longitudinal mode of conduction electron oscillations (**b**).

**Figure 2:**
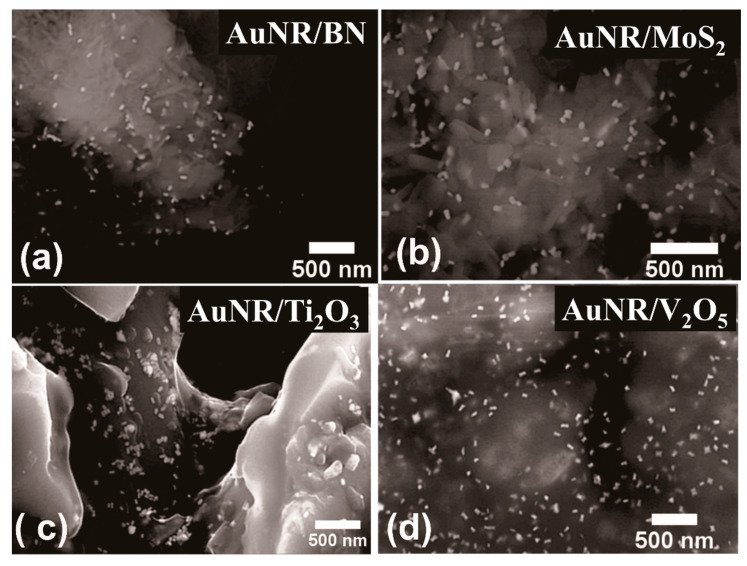
Scanning Electron Micrographs (SEM) from the as-synthesized short anisotropic gold nanorods (AuNRs) on different supports: (**a**) BN, (**b**) MoS_2_, (**c**) Ti_2_O_3_, and (**d**) V_2_O_5_ supports, respectively (scale bar: 500 nm). The respective Electron dispersive spectra (EDS) spectra and other supported AuNRs are shown in the Electronic Supporting Information (Figures S1 and S2).

The Scanning Electron Microscopy (SEM) analyzed surface morphology of the as-synthesized short anisotropic AuNRs synthesized via seeded approach is shown in Figure 1a. The corresponding length and width of the as-synthesized gold nanorods were calculated to be 52.02±5.84 nm and 20.73±2.49 nm, respectively. This corresponds to an aspect ratio of ~2.51. It is reported that the lengths, widths, and aspect ratios of the AuNRs can be varied upon variation in the respective contents present in the growth solution, namely the concentration of seeds, silver ions, and ascorbic acid or by addition of suitable additives to the growth solution, respectively [33]. The morphology and dimensions of the AuNRs as depicted in Figure 1a correspond to a specific value of the reactants present in the growth solution, which has been elaborated in the ESI. As can be observed from the SEM image, the majority of the as-synthesized nanoparticles were anisotropic AuNRs, with very low levels of other nanoparticle shapes, such as spherical or quasi-spherical nanoparticles. Figure 1b shows the optical absorbance spectra obtained from the as-synthesized AuNRs. The optical absorbance spectra from the as-synthesized AuNRs were primarily dominated by the presence of surface plasmons, which represent coherent oscillations of the Au 6s conduction electrons in resonance with the external electromagnetic radiation field. Surface plasmons from spherical AuNPs coupled well with the external electromagnetic radiation in the form of light in the visible range, thus exhibiting rich colors. The as-synthesized anisotropic AuNRs displayed two as such localized surface plasmon resonance (LSPR) peaks, located at ~512 nm and ~694 nm, respectively. These are due to the different modes of oscillation of the free conduction electrons, corresponding to the transverse and longitudinal dimensions of the AuNRs, respectively [34]. The longitudinal plasmon band in anisotropic AuNRs is sensitive to any changes in their aspect ratios, which also allows the directional routing of electromagnetic radiation in the near-infrared region [35]. The thiol-based linker molecule, thioglycolic acid, was used in this study to bridge the two materials, namely (1) the presynthesized short anisotropic AuNRs, and (2) different solid supports viz. TiO_2_, Al_2_O_3_, V_2_O_5_, Ti_2_O_3_, NiO, BN, and MoS_2_, respectively. The as-synthesized AuNRs were subject to purification in aqueous media prior to their functionalization with the thiol-based linker molecule, thioglycolic acid. The functionalization process of both the support materials and the as-synthesized purified short anisotropic AuNRs for their successful anchoring is described in the ESI. The scanning electron microscopic (SEM) images depicting the surface morphology of the supported AuNRs onto different substrates are shown in Figure 2.

**Figure 3:**
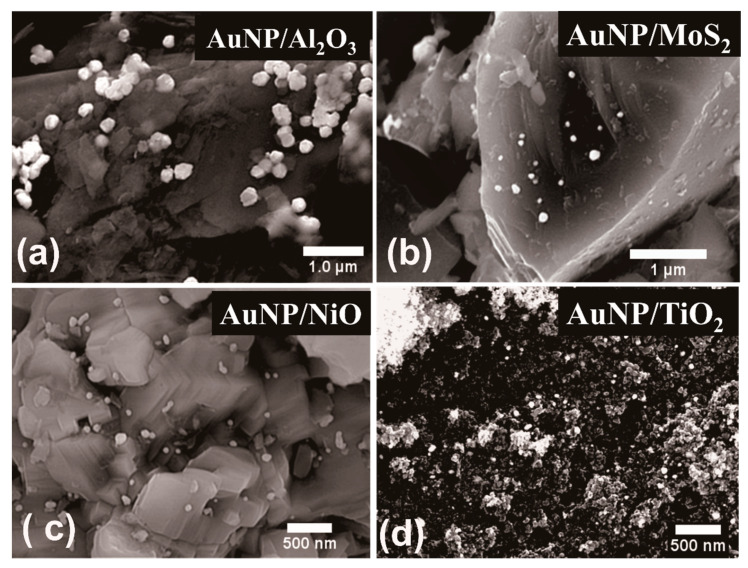
SEM images from gold nanoparticles (AuNPs) supported onto various substrates using the chemical impregnation synthesis route. The selected supports are (**a**) Al_2_O_3_, (**b**) MoS_2_, (**c**) NiO, and (**d**) TiO_2_, respectively. EDS from these supported AuNPs and AuNPs on other supports are shown in the Electronic Supplementary Information (Figures S1 and S3).

From the SEM images, it can be observed that the AuNRs were well dispersed over the support materials with minimized agglomeration. Most of the AuNRs were embedded inside the support materials or attached themselves with different orientations onto the solid supports, thereby exposing their partial lengths or widths to be imaged by scanning electron microscopy. The respective EDS spectra from each as-synthesized AuNRs supported onto these selected supports are shown in Figure 2, which reveal the characteristic elemental signals from both the support materials and AuNRs. In the chemical impregnation method, a strong reducing agent, sodium borohydride (NaBH_4_) was used to produce gold nanoparticles, which were subsequently attached onto the selected support materials. The detailed synthesis procedure can be found in the ESI. The supported AuNPs produced following this approach varied widely in size, as can be observed from Figure 3. The particle sizes varied from 9.46±1.8 nm to 212.94±19.92 nm, respectively, on different support materials. Because all synthesis conditions were fixed, this wide variation in size may have possibly resulted from the influence of support materials. The respective EDS spectra from each such catalysts also reveal the characteristic signals representative of both the AuNPs and the support materials.

### 3.1. Au 4f X-ray Photoelectron Spectroscopic Analysis from the Supported Gold Nanoparticles and Nanorods

**Figure 4:**
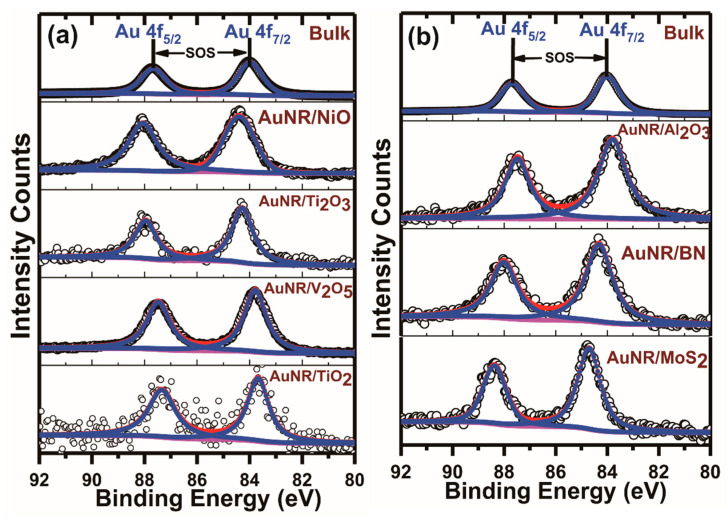
Fitted high-resolution X-ray Photoelectron Spectroscopic (XPS) Au4f spectra from the as-synthesized supported AuNRs on different materials: (**a**) NiO, Ti_2_O_3_, V_2_O_5_, TiO_2_, and (**b**) Al_2_O_3_, BN, MoS_2_ respectively. Au 4f spectra from two bulk gold samples are also shown for comparison purposes. The C 1s peak from adventitious carbon was selected as the reference peak and was used as a calibration standard for these supported nanoparticles. The spin-orbit-splitting (SOS) values were calculated from the separation between the two core level excitation peaks, Au 4f_7/2_ and Au 4f_5/2_, respectively. Contrary to the standard bulk reference values, the Au 4f_7/2_ peaks showed both upshifts and downshifts in presence of the support materials.

Common X-ray Photoelectron Spectrometers employ either monochromated or non-monochromated X-rays to eject core electrons from the depth of a few nanometers from the surface in a nondestructive manner. The photo-ejected electrons are then analyzed to obtain meaningful information on the core-level electronic structures from the catalyst samples under inspection. Most of the meaningful information is often obtained upon the proper analysis of the spectra. These include (1) the position of the binding energy peaks corresponding to the excitation of the core-level energy levels, representing the origin of the photo-ejected electrons; (2) the photoelectron peak intensities, representing the photoelectric cross section and escape depth; (3) the peak linewidth, represented by a Lorentzian profile relating to the core-hole lifetime; and (4) the distance of separation between the peaks related to specific subshells $\left(j_{\pm }=l\pm \frac{1}{2}\right)$, separated by spin-orbit coupling effects. A typical Au 4f XPS spectrum features two photoemission peaks corresponding to the core-level Au 4f_7/2_ and Au 4f_5/2_ excitations, respectively (Figure 4). In bulk gold, these two excitation peaks are well separated by a distance of 3.67 eV, with the Au 4f_7/2_ binding energy peak position appearing at 84.00 eV [36]. In AuNP-based catalyst materials, any deviation in the Au 4f_7/2_ binding energy peak position from this standard bulk value is often seen as a change in the surrounding chemical environment or electronic states of the AuNPs. This is evident in all our catalyst samples, where either an upshift or downshift in the Au 4f_7/2_ binding energy position relative to the bulk standard values was observed. However, irrespective of such wide variations in the Au 4f_7/2_ binding energy peak positions in the catalyst samples, the metallic Au^0^ character of the AuNPs/AuNRs was retained. This is evidenced by (1) the constant spin-orbit splitting value, ~3.67 eV, i.e., the separation binding energy values between Au 4f_7/2_ and Au 4f_5/2_ core-level excited photoelectron lines in all the AuNP/AuNR-based catalyst samples, which matched very well with the spin-orbit-splitting values from bulk reference gold samples; and (2) the nonoccurrence of any peaks at binding energy peak positions at 85.8 eV and 89.1 eV, which are the characteristic signals from Au^3+^. It is also well documented that in many AuNP-based catalysts, the Au 4f_7/2_ binding energy position of the fully reduced gold species range from 82.9 eV to 84.5 eV [26]. Therefore, although the metallic character of gold in both AuNPs and AuNRs was retained in all the catalyst samples, the presence of the support materials altered the Au 4f_7/2_ binding energy positions to vary in both ways, which is an indication of interaction of the AuNPs/AuNRs with the selected support materials. We further observed that the supported AuNP-based catalysts prepared by the chemical -impregnation method displayed Au 4f_7/2_ binding energy positions at values lower in magnitude by 0.1–0.15 eV compared against the supported AuNR-based catalysts prepared by surface functionalization synthesis. This could be due to the difference in synthesis procedures or pretreatment conditions. Moreover, apart from the small magnitude of binding energy differences in the Au 4f_7/2_ peak position, both the upshift and downshift trends remained similar in respective support materials, irrespective of both the synthesis routes.

### 3.2. Au 4f_7/2_-to-Au 4f_5/2_ Peak Intensity and Linewidth Ratios in the Supported AuNPs, AuNRs and Bulk Gold

Following the spectral analysis of the Au 4f_7/2_ binding energy positions in all our supported AuNP/AuNR-based catalysts, which showed deviations from the bulk gold standard reference values, we further analyzed the Au 4f doublets from all the catalysts. These doublet patterns resulted from the spin-orbit coupling effects, following different $j_{\pm }=\left(l\pm s\right)$ values corresponding to the Au 4f_7/2_ and Au 4f_5/2_ excitations appearing in high resolution Au 4f XPS spectra (Figure 4). The integrated XPS peak intensities under each spin-orbit component represent the degeneracy or multiplicity values $\left(2j_{\pm }\;+\;1\right)$ associated with the respective subshells. So, following a $\left(2j_{\pm }\;+\;1\right)$ rule, the Au 4f_7/2_-to-Au 4f_5/2_ peak intensity ratios in a high-resolution Au 4f XPS spectrum are normally assumed to follow an 8:6 (or 4:3) ratio or, more commonly, a standard statistical value of 1.33. We therefore made a thorough analysis of the Au 4f_7/2_ and Au 4f_5/2_ peak areas based on our peak fitting results.

**Figure 5:**
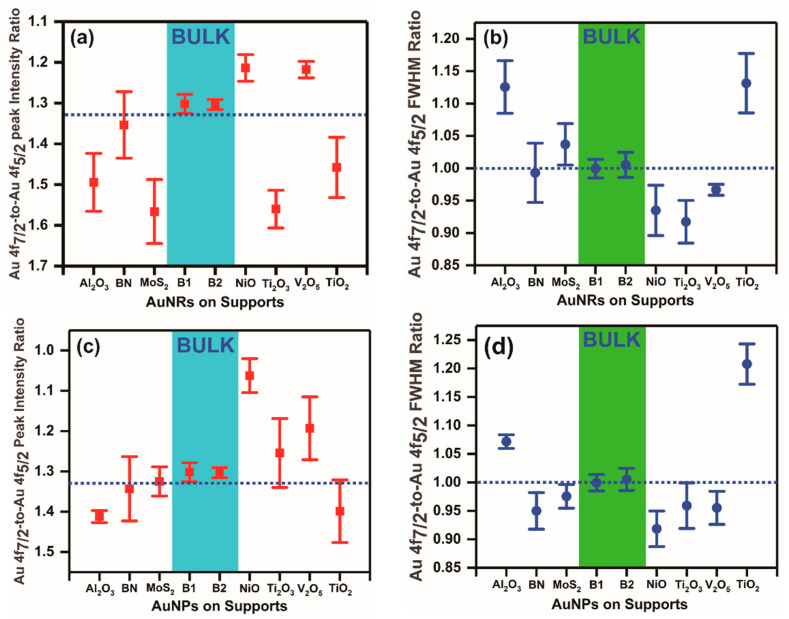
The Au 4f_7/2_-to-Au 4f_5/2_ peak intensity (**a**,**c**) and linewidth ratios (**b**,**d**) in the supported gold nanoparticles and gold nanorods. A comparison is made in all cases against bulk gold samples, B1 and B2, in all cases. Both the Au 4f_7/2_-to-Au 4f_5/2_ peak intensity and linewidth ratios deviated largely from bulk standard values.

We observed that in the two bulk planar gold samples, the Au 4f_7/2_-to-Au 4f_5/2_ peak intensity ratios remained closer to 1.33 (1.32±0.02) (Figure 5a,c), while the scenario was very different in the supported AuNP/AuNR catalysts. In TiO_2_-supported small gold nanoparticles, the Au 4f_7/2_-to-Au 4f_5/2_ peak intensity ratios were observed to vary in a wide range, from 1.09±0.04 to 1.63±0.08, respectively [31]. It is now well established that the catalytic activities are largely influenced by the sizes of the nanoparticles, nature of support materials, and synthesis conditions. In this study, the supported AuNPs synthesized via chemical impregnation synthesis exhibited sizes in a wide range, from 9.46±1.8 nm to 212.94±19.92 nm, on different support materials. As such, these particle sizes are much larger than previous reports [31,32]. The Au 4f_7/2_-to-Au 4f_5/2_ peak intensity ratios in such large supported gold nanoparticles synthesized via chemical impregnation procedures demonstrated varied values in a wide range, from 1.06±0.04 to 1.41±0.06, respectively (Figure 5c). This indicates that the Au 4f_7/2_-to-Au 4f_5/2_ peak intensity ratios deviate strongly from the standard statistical bulk value, 1.33. Furthermore, such observations also demonstrated that the Au 4f_7/2_-to-Au 4f_5/2_ peak intensity ratios are not limited to occur in small supported gold nanoparticles only. Moreover, the deviations also occurred in as many different types of support materials, namely oxides, dichalcogenides, and nitrides, thereby further demonstrating that they are not only limited to TiO_2_ supports. Upon extension of our analysis to the supported AuNR-based catalysts, which were synthesized by a different synthesis route, we also observed similar deviations in the Au 4f_7/2_-to-Au 4f_5/2_ peak intensity ratios from the bulk standard statistical value. The Au 4f_7/2_-to-Au 4f_5/2_ peak intensity ratios in the as-synthesized supported AuNRs-based catalysts varied widely, from 1.21±0.03 to 1.57±0.08, respectively (Figure 5a). Therefore, similar to the variations in Au 4f_7/2_ binding energy positions from the bulk standard values, the Au 4f_7/2_-to-Au 4f_5/2_ peak intensity ratios also varied widely from the standard statistical multiplicity ratio, 1.33, irrespective of synthesis conditions, sizes of the nanoparticle, variation in the morphologies of the nanoparticles, or choice over the support materials. In contrast, the Au 4f_7/2_-to-Au 4f_5/2_ peak intensity ratios in the two bulk planar gold samples did not display any such wide variations from the standard statistical multiplicity ratio, 1.33 (Figure 5a,c).

Following our analysis of the Au 4f_7/2_ binding energy position and Au 4f_7/2_-to-Au 4f_5/2_ peak intensity ratios in all the supported AuNP/AuNR catalyst samples, which displayed deviations from the standard bulk values, we analyzed the Au 4f_7/2_-to-Au 4f_5/2_ linewidth ratios in all the catalyst samples. For comparison purposes, we also analyzed the Au 4f_7/2_-to-Au 4f_5/2_ linewidth ratios in the two bulk standard samples. As mentioned earlier, our catalyst samples varied in size, morphology, and synthesis conditions. The calculated Au 4f_7/2_-to-Au 4f_5/2_ linewidth ratios in the supported AuNPs prepared by the chemical impregnation procedure displayed variations similar to both Au 4f_7/2_ binding energy positions and Au 4f_7/2_-to-Au 4f_5/2_ peak intensity ratios, respectively. The Au 4f_7/2_-to-Au 4f_5/2_ linewidth ratios varied from 0.92±0.031 to 1.21±0.035 in such supported AuNPs prepared by the chemical impregnation synthesis procedure (Figure 5d). In the supported AuNRs synthesized by the surface functionalization synthesis procedure, the Au 4f_7/2_-to-Au 4f_5/2_ linewidth ratios also displayed similar variations from 0.92±0.033 to 1.13±0.045, respectively (Figure 5b). The Au 4f_7/2_-to-Au 4f_5/2_ linewidth ratios therefore varied widely from the standard statistical bulk values in the supported AuNP/AuNR-based catalysts under different experimental conditions, namely the synthesis procedure, sizes and shapes, morphologies, and support materials, respectively. On the other hand, the Au 4f_7/2_-to-Au 4f_5/2_ linewidth ratios in the two bulk standard planar gold samples did not exhibit any such wide variations. The calculated Au 4f_7/2_-to-Au 4f_5/2_ linewidth ratios in the two bulk planar gold samples were observed to be 1.001±0.014 (B1) and 1.005±0.019 (B2), respectively (Figure 5b,d).

**Figure 6:**
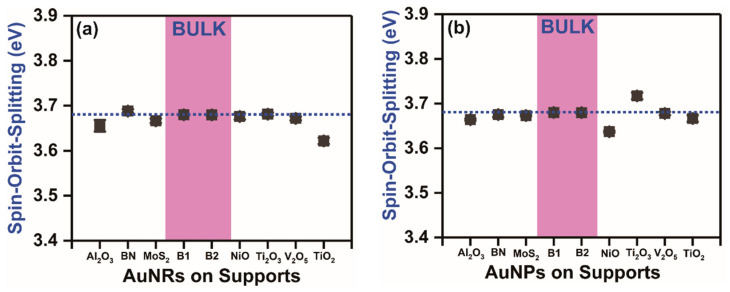
Spin-orbit-splitting values calculated for gold nanorods (AuNRs) on different supports (**a**) and gold nanoparticles (AuNPs) on different supports (**b**). The spin-orbit-splitting values in both supported gold nanoparticles and gold nanorods are compared against bulk gold (B1 and B2) values in all cases. Unlike the Au 4f_7/2_-to-Au 4f_5/2_ peak intensity and linewidth ratios, the spin-orbit-splitting values in all supported AuNRs and AuNPs remained closer to atomic or bulk standard values.

In addition to all the above observations, namely the Au 4f_7/2_ binding energy position, Au 4f_7/2_-to-Au 4f_5/2_ intensity ratios, and Au 4f_7/2_-to-Au 4f_5/2_ linewidth ratios, we looked closer into the calculated spin-orbit-splitting values in all the catalyst samples (Figure 6a,b). In contrast to all the above observations, the calculated spin-orbit-splitting values in all the supported AuNP/AuNR-based catalyst samples did not display any such wide variations. The spin-orbit-splitting values in the supported AuNPs prepared using chemical-impregnation procedure varied from 3.64 eV to 3.72 eV, whereas the supported AuNRs prepared by surface-functionalization synthesis route varied from 3.62 eV to 3.69 eV, respectively (Figure 5a,b). In both the cases, the average spin-orbit-splitting values (3.67±0.02 eV) remained closer to the atomic or bulk values [37,38].

In many AuNP-based catalyst materials, the Au 4f_7/2_ binding energy position often displays variations from its original bulk counterpart, 84.0 eV. In our case, while the two bulk planar gold samples displayed binding energy values closer to the standard bulk reference value, 84.0 eV, the presence of support materials resulted in both upshift and downshift in the Au 4f_7/2_ standard binding energy value. In order to understand the anomalous features observed in the Au 4f core-level degeneracy or multiplicity ratios, we resorted to the relativistic treatments on gold chemistry. The reason is that gold is the most relativistic element in the periodic table with Z < 100 [39]. Many of the unique physicochemical properties of gold, viz. yellowish appearance and nobleness, high electron affinity, higher oxidation potentials, small coordination numbers, strongly reduced atomic, and ionic radii, have been quantitatively well reproduced in relativistic treatments [40,41]. On the other hand, nonrelativistic theoretical calculations on physical parameters of gold, such as the first ionization potential, electron affinity, and interband energy between the top 5d band and the Fermi level energy in the half-filled 6s band values, exhibit greater deviations from experimental values than when treated in relativistic frameworks [39]. This further indicates a deep probe into relativistic gold chemistry, in which two direct effects emerge: (1) The spin-orbit coupling effect, causing the Au 4f orbitals to split into Au 4f_7/2_ and Au 4f_5/2_ subshells; and (2) expansion of the outermost *d* and *f* orbitals, owing to the relativistic contraction of the *s* and *p* orbitals [42]. Relativistic contraction of Au 6s orbitals overlapping with the expanded Au 5d orbitals results in strong *d-d* interactions and *s-d* hybridized orbitals. The *sd* hybridized orbitals are expected to increase the number of free *d*-states (or *d*-holes). This effect is prominent in nanoparticles, which expose a higher number of undercoordinated surface atoms in comparison to bulk gold. The reduction in the coordination numbers in the surface atoms leads to more discrete-like narrow *d*-bands. This reduction in the *d*-delocalization and *sd* hybridization in AuNPs/AuNRs reduce the true *d*-electron counts in AuNPs/AuNRs. The *5d-(6s-6p)* hybridization occurs when employed in a relativistic tight binding band model for gold. The relativistic emission intensity from a core level $n^{\prime }l^{\prime }j^{\prime }\left(j^{\prime }=l^{\prime }\pm \frac{1}{2}\right)$ for a dipole transition is [43]

$$I_{n^{\prime }l^{\prime }j^{\prime }}\left(\omega \right)\propto \left(2j^{\prime }+1\right)\underset{lj}{\sum }A_{lj}^{n^{\prime }l^{\prime }j^{\prime }}\left(E\right)N_{lj}\left(E\right)$$

where $A_{lj}^{n^{\prime }l^{\prime }j^{\prime }}\left(E\right)$ are slowly varying functions of energy and $N_{lj}\left(E\right)$ represents the conduction-band density of states. For 4*f* core-level transitions involving spin-orbit-split $j^{\prime }=\frac{7}{2}$ (Au 4f_7/2_) and $j^{\prime }=\frac{5}{2}$ (Au 4f_5/2_) excitations, the ratio of the integrated intensities is [43]

$$\frac{I_{4f_{\frac{7}{2}}}}{I_{4f_{\frac{5}{2}}}}=\frac{20h_{\frac{5}{2}}}{h_{5/2}+21h_{3/2}}$$

where $h_{5/2}$ and $h_{3/2}$ are the hole densities in the *d* band.

This relativistic expression therefore implies that a decrease in the number of true *d* electron counts owing to *sd* hybridizations affected the photoemission peak intensities from the core-levels. Thus, the Au 4f_7/2_-to-Au 4f_5/2_ peak intensity ratios in the supported AuNP/AuNR catalysts vary in a wide range and deviate from the standard statistical bulk gold values.

In an XPS spectrum, the photoelectron peak linewidths represent the core-hole lifetimes associated with the representative subshell from which a photoelectron is ejected. Following the ejection of the core-electron, the excited atom undergoes through a cascade of radiative and nonradiative decay processes. The individual spin-orbit components in a typical photoemission process undergo similar decay processes with equal decay rates. This is the case in the two bulk gold samples which display equal Au 4f_7/2_-to-Au 4f_5/2_ linewidth ratios. An unequal Au 4f_7/2_-to-Au 4f_5/2_ linewidth ratio therefore indicates the presence of additional decay channels facilitating the de-excitation processes. In metallic systems, this additional decay channels often involves Coster–Kronig electron transitions, even with very small spin-orbit splittings [44]. This is because of the free conduction electrons in the metallic systems. A higher 4f_7/2_-to-4f_5/2_ peak linewidth ratios or increased 4f_7/2_ linewidth implies an enhanced Auger decay rate, which has been observed in W (110) and Ta (110) surfaces and has been postulated to occur in many metallic elements where conduction electrons dominate the core-hole decay [45,46]. The shorter 4f_7/2_ core-hole lifetime or increased linewidth has been attributed to be due to an N_7_O_45_O_45_ Auger transition. An increase in linewidth of the deeper $J_{-}\left(=l-s\right)$ spin-orbit component was observed in Ir 4d_3/2_ and Au 4d_3/2_ [44]. The broadening of the deeper Au 4f_5/2_ spin-orbit component or lower Au 4f_7/2_-to-Au 4f_5/2_ peak FWHM ratios in our supported AuNPs/AuNRs therefore indicates the possibility of additional decay channels. An increased 4f_5/2_ linewidth was observed in the Ta (111), W (111), and W (100) surfaces, which was attributed to the N_6_N_7_O_45_ super Coster–Kronig Auger decay processes [47]. Similarly, an increase in the 4f_5/2_ linewidth in Hf was attributed to the presence of an N_6_N_7_O_45_ Coster–Kronig decay channel [48], which was also recently proposed [31] in TiO_2_-supported small gold nanoparticles.

Contrary to all the observed deviations from standard statistical values, the spin-orbit-splitting values remained almost constant in both the supported AuNP and AuNR-based catalysts, which was also observed to be similar in bulk planar gold samples. This indicates that the spin-orbit-splitting values were unaffected by any change in particle size, shape, morphology, synthesis conditions, and presence of any support materials. This constant nature of the spin-orbit-splitting values in both the supported AuNPs and bulk Au therefore confirm that it is an ideal standard fitting parameter in high-resolution core-level Au 4f spectra from Au-based catalyst materials.

## 4. Conclusions

Au 4f core-level spin-orbit components from a set of gold nanoparticle catalysts supported on different substrate materials were analyzed carefully. The interaction between the gold nanoparticles (AuNPs) and short anisotropic gold nanorods (AuNRs) with the support materials was evidenced from the core-level shifts in the Au 4f_7/2_ binding energy positions. Like variations in the Au 4f_7/2_ binding energy positions, the Au 4f_7/2_-to-Au 4f_5/2_ peak intensity and linewidth ratios displayed deviations from the standard statistical ratios. These deviations occurred in all supported AuNP-based catalyst samples, irrespective of synthesis conditions, shape, size, and morphology of the gold nanoparticles and support materials. On the other hand, irrespective of any such changes, the spin-orbit-splitting values did not show such wide variations and matched well with the atomic and bulk standard values. The more localized 5d electrons in narrowed *d*-bands, reduced *s-d* hybridizations, higher number of undercoordinated surface atoms, influence from the support materials, relativistic photoemission, and existence of additional Coster–Kronig decay channels could be the origin of the observation of such anomalous features in the Au 4f core-level spin-orbit components from various supported gold nanostructures presented in this study. The constant nature of the Au 4f spin-orbit-splitting value, irrespective of any such varied experimental conditions, demonstrates its importance as an ideal peak fitting parameter in both the supported AuNP-based catalysts and bulk gold as well. We envisage the existence of such anomalous features to be probed in other supported nanoparticle-based catalysts to shed more light on such deviations from the bulk standard statistical multiplicity ratios.

## Data Availability

Data is contained within the article or in Electronic Supporting Information (ESI).

## Supplementary Materials

The following are available online at https://www.mdpi.com/2079-4991/11/2/554/s1, pp S1: Synthesis of gold nanoparticles on various supports, pp S1: Synthesis by Chemical-Impregnation procedure, pp S2: Synthesis of supported gold nanorods onto the selected supports, pp S2: Synthesis of CTAB atabilized Gold Seeds, pp S2: Synthesis of gold nanorods (AuNRs), pp S3: Support of the pre-synthesized AuNRs onto the selected supports, Figure S1: Small anisotropic gold nanorods (AuNRs) and gold nanoparticles (AuNPs) supported on NiO, Al_2_O_3_, BN, and V_2_O_5_ respectively, Figure S2: Electron Dispersive Spectra (EDS) from the as-synthesized supported gold nanorods (AuNRs), Figure S3: EDS spectra from AuNPs supported on various substrates.
