# Supplementary material for: Spin-Orbit Coupling Effects in Au 4f Core-Level Electronic Structures in Supported Low-Dimensional Gold Nanoparticles
Source: Nanomaterials (Basel). 2021 Feb 23;11(2):554. doi: 10.3390/nano11020554 (PMC7926876; doi:10.3390/nano11020554)
Supplement: Supplementary file 1 [file nanomaterials-11-00554-s001.pdf]

Supporting Information

Spin Orbit Coupling Effects in Au 4f Core-level Electronic Structures in Supported Low-Dimensional Gold Nanoparticles

Synthesis of gold nanoparticles on various supports

For the synthesis of supported gold nanoparticles and gold nanorods, the following materials were used: Tetrachloroauric(III) acid ($HAuCl_4.3H_2O$, 99.995%), silver nitrate ($AgNO_3$, 99%), cetyltrimethylammonium bromide (CTAB, 98%), sodium borohydride ($NaBH_4$, >98%), ascorbic acid (AA, 99%), thioglycolic acid (TGA a.k.a. mercaptoacetic acid, 70 wt%), V_2O_5 (divanadium pentoxide, 99.6%), and Al_2O_3 (Alumina) were obtained from Sigma-Aldrich, Burlington, MA, USA, and used as received. MoS_2 (99%, Alfa-Aesar, Lancashire, UK), Boron Nitride (BN, 98.5%, National Nitride Technologies, Taichung, Taiwan), Ti_2O_3 (99.9%, Aldrich), and NiO (99%, Alfa-Aesar) were used as received. TiO_2 Degussa P25 (Nanoshell LLC, Willmington, DE, USA) containing 70% anatase and 30% rutile was used in the experiment. Millipore DI water (Resistivity: 18.2 MΩ) was used throughout the synthesis and purification processes.

All the glassware was cleaned with Millipore DI water (Resistivity: 18.2 MΩ), ethanol and acetone multiple times prior to their first use in order to avoid possible contaminants during the nanoparticle synthesis process. Two different synthesis routes, namely: (1) Chemical-Impregnation and (2) Surface-Functionalization methods were adopted for the preparation of supported gold nanoparticles and gold nanorods respectively.

(1) Synthesis by Chemical-Impregnation procedure

In a typical synthesis procedure, respective amounts of the support materials [MoS_2 (0.024 g); Al_2O_3 (0.015 g); BN (0.010 g); NiO (0.011 g); Ti_2O_3 (0.022 g); V_2O_5 (0.027 g) and TiO_2 (0.012 g)] were added to a 15ml aqueous solution containing $HAuCl_4$ at 9×10^{-4}M. The mixture solution was then stirred for 3 h under dark condition. Next, 0.6 mL of a freshly prepared ice-cold $NaBH_4$ [0.1 M] solution was added dropwise to the mixture solution containing supports and $HAuCl_4$, under rapid stirring condition. The stirring was further continued for 6 h under dark environment. The solution was then centrifuged to collect the precipitates. The precipitates were washed multiple times with de-ionized water and centrifuged to obtain the final products. The final products were then oven dried at 95 °C for 16 h to obtain the catalyst samples. The solid catalysts were then ground in an agate mortar to obtain fine powders for their further characterizations.

(2) Synthesis of supported gold nanorods onto the selected supports

This procedure involves two important steps, namely: (1) Synthesis of gold nanorods (AuNRs) via a two-step seed mediated approach, and (2) support of the pre-synthesized AuNRs onto the selected supports via a surface functionalization synthesis route. The two-step seed-mediated approach synthesis of AuNRs requires (i) Synthesis of small-sized CTAB capped gold seed particles, and (ii) Preparation of a growth solution for the AuNR growth using the pre-synthesized seeds.

(i) Synthesis of CTAB stabilized Gold Seeds:

For the synthesis of CTAB stabilized gold seeds, 0.25 mL of $HAuCl_4$ [10 mM] was added to an aqueous solution containing 7.5 mL of CTAB [0.1 M]. The solution was gently mixed and stirred afterwards. Then, 0.6 mL of a freshly prepared ice-cold $NaBH_4$ (10 mM) was rapidly added to the solution under vigorous stirring condition. The vigorous

stirring was continued for further 3 h to consume all of $NaBH_4$ contents present in the solution.

(ii) Synthesis of gold nanorods (AuNRs):

For the synthesis of AuNRs, a growth solution containing CTAB, $HAuCl_4$, $AgNO_3$ and ascorbic acid (AA) was prepared at first. First, 6 mL of $HAuCl_4$ [10 mM] was added to 142.5 mL of CTAB [0.1 M] followed by gentle mixing. Next, 0.96 mL of ascorbic acid [0.1 M] was added to the solution. The addition of AA resulted in a colorless appearance of the solution, indicating the reduction of Au^{3+} to Au^{+}. 0.9 mL of $AgNO_3$ [10 mM] was added to the solution afterwards and gently mixed. Finally, 0.1 mL of the pre-synthesized seeds were added to the solution to initiate AuNR growth. The solution was gently mixed for ~10 s during each addition interval. Finally the solution was kept undisturbed for at least 3 h for the complete formation of AuNRs.

(iii) Support of the pre-synthesized AuNRs onto the selected supports:

The as-synthesized AuNRs were purified via centrifugation method many times so as to remove excess of CTAB before their functionalization with TGA. For their functionalization with TGA, the purified AuNRs were added dropwise to an aqueous solution containing 30 mL TGA [5×10^{-1} M] under stirring condition. After the complete addition of the AuNRs, the solution was kept under stirring for more 3 h. In a different experiment, specific amounts of the supports [BN (0.3 g); V_2O_5 (2.18 g); NiO (0.9 g); Al_2O_3 (1.22 g); MoS_2 (1.9 g); Ti_2O_3 (1.73 g) and TiO_2 (0.96 g)] were added to the aqueous solution containing 40 mL TGA [5×10^{-1} M] under stirring condition for 3 h. Finally, the TGA-functionalized AuNRs were added dropwise to the functionalized supports while stirring. After complete addition, the solution was further stirred for 6 h. The solution was then centrifuged to obtain the solid precipitates, washed with de-ionized water to remove excess of TGA, and finally oven-dried at 95 °C for 16 h to obtain the solid

precipitates. The oven-dried catalysts were then ground in an agate mortar to obtain fine powders for their subsequent characterizations.

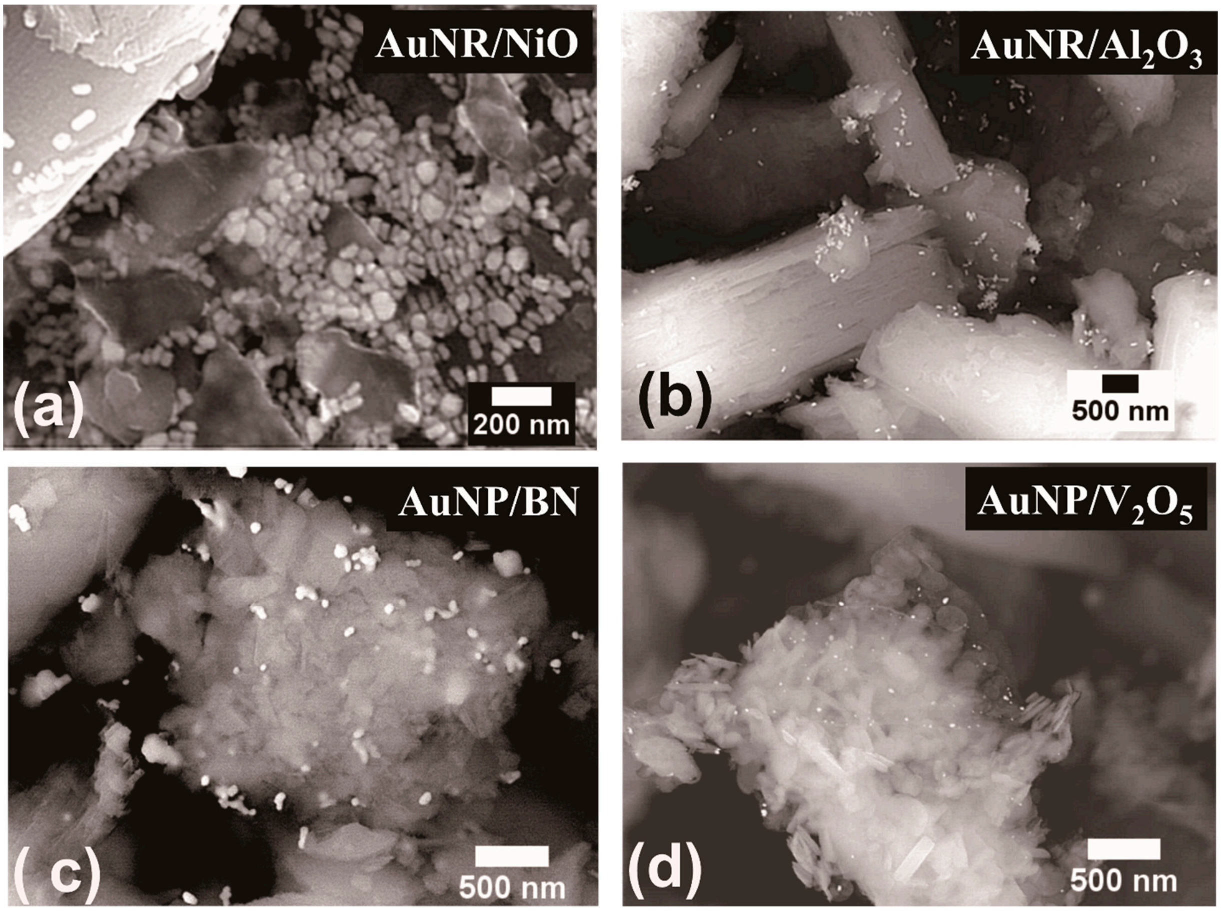

Figure S1: Small anisotropic gold nanorods (AuNRs) and gold nanoparticles (AuNPs) supported on (**a**) NiO, (**b**) Al_2O_3, (**c**) BN, and V_2O_5 respectively. The short anisotropic AuNRs were synthesized by a two-step seeded approach and were functionalized to anchor on the support materials. Chemical impregnation synthesized AuNPs were supported on the selected materials.

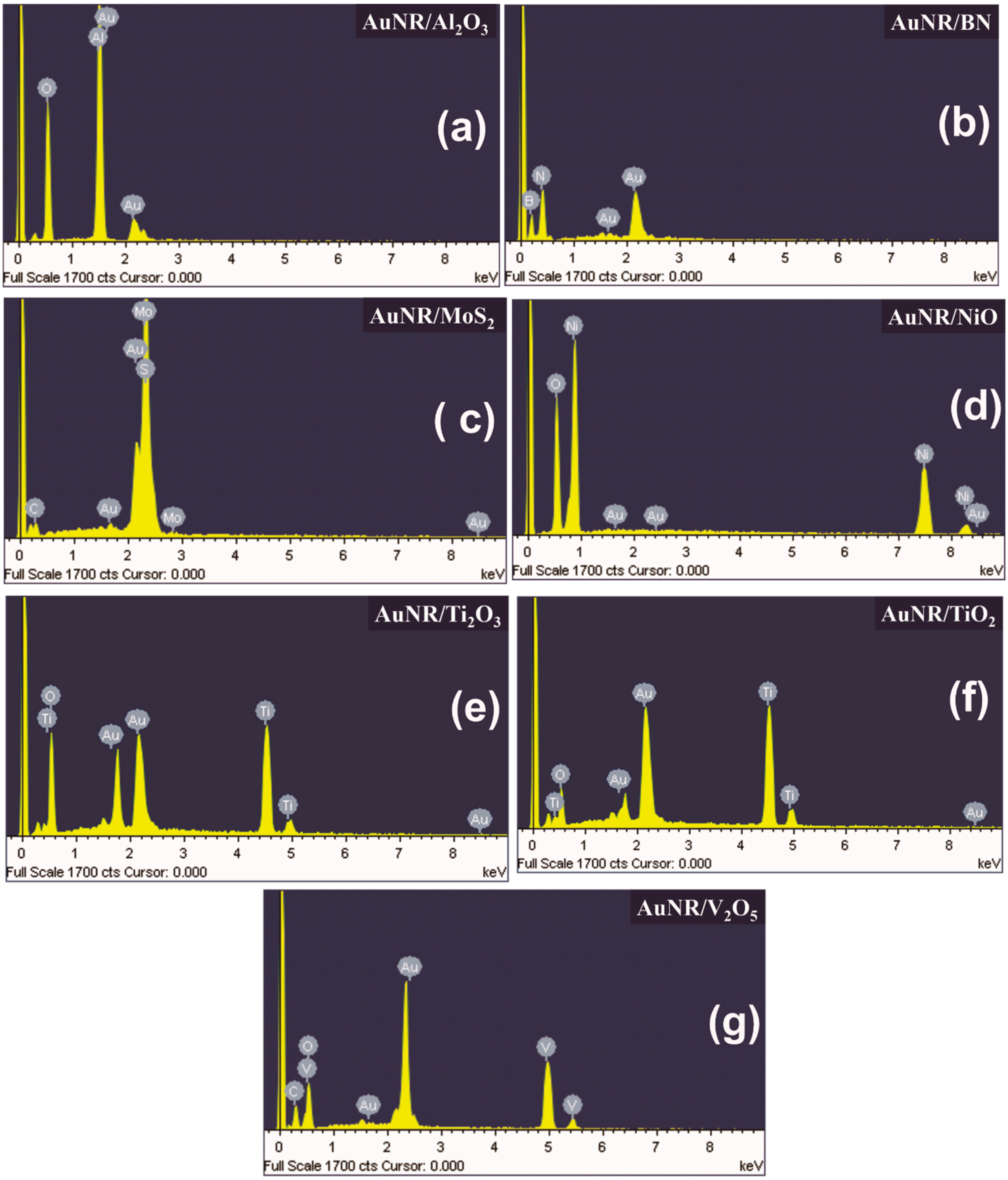

Figure S2: Electron Dispersive Spectra (EDS) from the as-synthesized supported gold nanorods (AuNRs). Presence of AuNRs and the support materials is evidenced from the spectra. Gold nanorods (AuNRs) supported on (**a**) Al_2O_3, (**b**) BN, (**c**) MoS_2, (**d**) NiO, (**e**) Ti_2O_3, (**f**) TiO_2, and (**g**) V_2O_5 respectively.

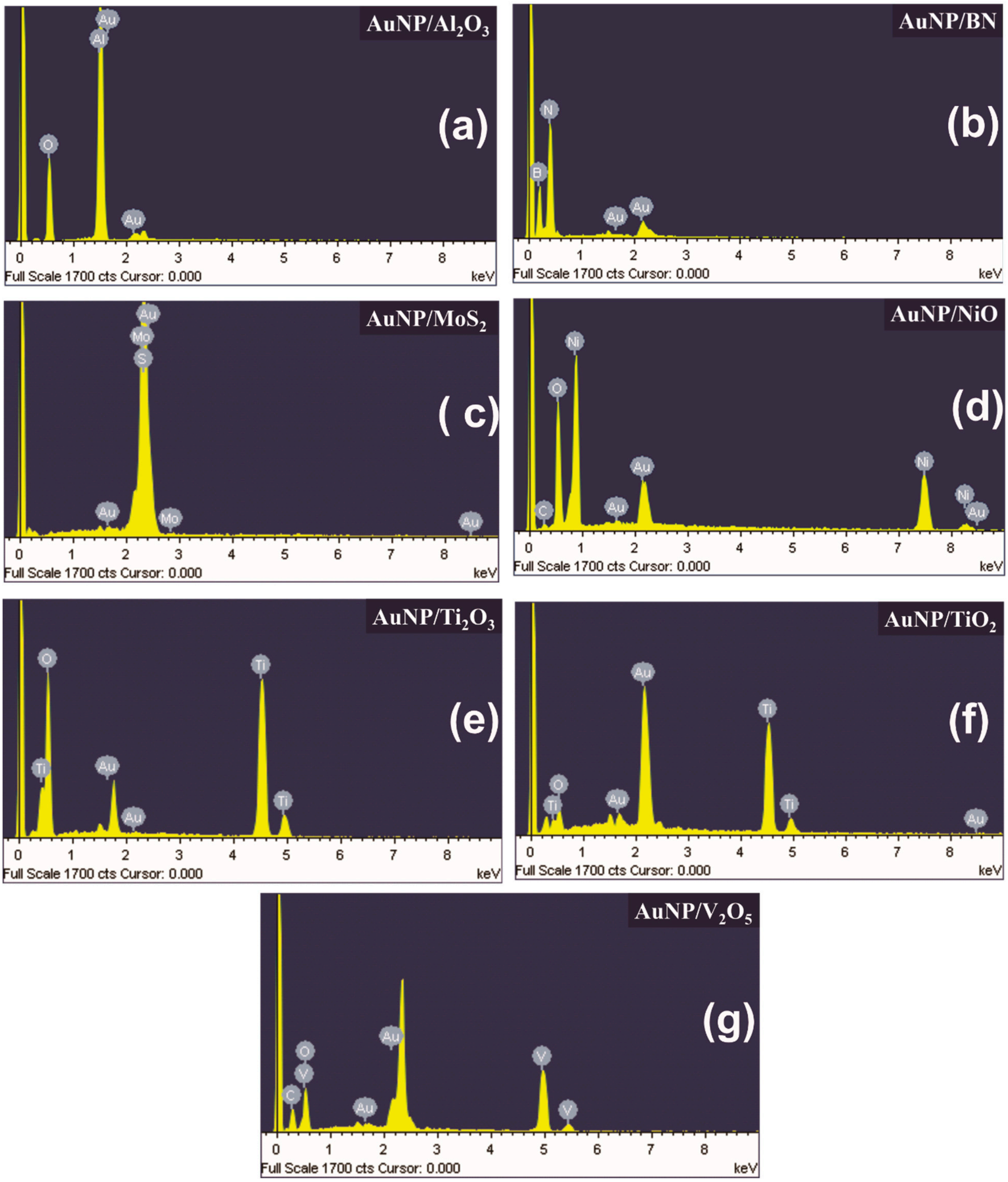

Figure S3: The elemental information from the support materials and gold nanoparticles (AuNPs) prepared by chemical impregnation synthesis route is evidenced from their respective EDS spectra. EDS spectra from AuNPs supported on (**a**) Al_2O_3, (**b**) BN, (**c**) MoS_2, (**d**) NiO, (**e**) Ti_2O_3, (**f**) TiO_2, and (**g**) V_2O_5, respectively.
